# The effect of tennis batting sound on anxiety: a randomized controlled trial and basic acoustic analysis

**DOI:** 10.3389/fpsyg.2023.1233599

**Published:** 2023-12-07

**Authors:** Hao Wang, Geng Zhang, Xiang Li, Shihong Pu

**Affiliations:** School of Physical Education, China West Normal University, Nanchong, China

**Keywords:** sports psychology, tennis, anxiety, voice, mental health

## Abstract

**Purpose:**

To investigate the potential role of the unique sound of tennis in alleviating anxiety. Previous research has consistently shown that exercise can mitigate anxiety, primarily attributed to the impact of increased physical activity on hormonal and neurostructural changes. However, in daily life we find that one of the reasons people are drawn to tennis is its distinctive sound. In this study, we specifically examined the influence of this sound on anxiety.

**Methods and results:**

In a randomized controlled experiment involving 96 participants reporting chronic anxiety (*n*_1_ = *n*_2_ = 48), we found that the control group exhibited an average reduction of 0.00156 in anxiety scores 4 weeks before and after the study. On the other hand, the experimental group, exposed to tennis stroke sound stimuli, showed an average reduction of 0.02896 in anxiety scores after 4 weeks, with some individuals even experiencing a decrease from anxiety to mild anxiety. Furthermore, the analysis of sound data revealed that the sound of tennis exhibited a pleasing timbre, with the primary sound frequencies ranging from 100 to 2,800 Hz. The rhythm of the sound had an average interval of approximately 1.758″ (± 0.41), corresponding to speed of approximately 93.6 km/h. The sound exhibited a steady rhythm, orderly variations in pitch, and a soothing timbre.

**Conclusion:**

This study confirms that the sound of tennis alone contributes to anxiety relief, attributed to its suitable loudness, steady rhythm, and orderly variations in pitch, all of which align with human auditory characteristics. This indicates that a considerable portion of the anxiety-alleviating effects of tennis attributed to its comforting sound.

## 1 Introduction

Anxiety is a complex emotional state characterized by apprehension, fear, tension, distress, and hyper vigilance, palpitations, sweating, and possibly intense panic (Rynn and Brawman-Mintzer, 2004). Research has shown that moderate levels of anxiety can increase an individual’s effectiveness and potential, as Harvard psychologists Robert Yerkes and John Dodson hypothesized in 1908 that maintaining a certain level of “alertness” can enhance performance (Jie-Lin and He-Zhan, 2015). However, excessive anxiety can have many negative effects, impairing attention, memory, and perceptual-motor functions (Eysenck et al., 2007; Moran, 2016; Runswick et al., 2018). If severe and prolonged anxiety is not effectively relieved, it can develop into an anxiety disorder (AD). Once anxiety has progressed to an anxiety disorder, it should be treated in conjunction with a doctor, typically with medication, physiotherapy, and psychological support (Testa et al., 2013). However, for overall human health, prevention is always better than cure. Reasonable relief of anxiety is essential in the prevention of anxiety disorders.

There is a significant body of research indicating that exercise has a positive impact on mental health. Exercise has been found to be effective in alleviating symptoms of anxiety and depression, reducing stress, improving mood and self-esteem, and enhancing cognitive function. Exercise has also been shown to have a protective effect against the development of mental health disorders.

Research on exercise and mental health has been highly accomplished in the fields of psychology, applied psychology, sport psychology, and psychiatry, with a large number of studies showing that exercise is effective in alleviating negative emotions (Stubbs et al., 2017) and enhancing mental health (Mead et al., 2008; Cooney et al., 2013; Hwang et al., 2019; Firth et al., 2020). However, most studies focus on the physiological changes brought about by exercise, such as changes in brain structure and related hormone levels. These studies have shown that exercise improves brain structure and has a beneficial remodeling effect on brain structures that control emotions, including the hippocampus (Hendrikse et al., 2022), prefrontal cortex, anterior cingulate cortex (Colcombe et al., 2003; Canbeyli, 2013), and amygdala (Duman et al., 1999; Lin et al., 2015). Furthermore, exercise has been found to boost the body’s hormone levels, such as dopamine (Shimojo et al., 2019), endorphins (Hildebrandt et al., 2014; Schoenfeld and Swanson, 2021), 5-hydroxytryptamine (Hu et al., 2015), norepinephrine (Goddard et al., 2010), and other hormones. In short, changes in hormone levels brought about by exercise have a significant impact on human mood (Wegner et al., 2014; Szuhany et al., 2015). These physiological changes are important factors influencing emotions and facilitating the alleviation of anxiety or depression.

Much of the existing research has focused on the emotional impact of physiological changes resulting from increased intensity of physical activity. However, little attention has been given to whether the sounds produced during exercise can influence mood. Tennis, a popular and sophisticated sport played around the world, is known for its distinctive tone and rhythm. The ITF Global Tennis Report 2021 indicates that as of 2021, more than 87 million people have participated in tennis across 41 surveyed countries, which represents a 4.5% increase compared to 2018. The North America region has the highest percentage of its population playing tennis (6.5%). Tennis enthusiasts in other countries are also steadily increasing. Undoubtedly, tennis, as a prevalent sport worldwide, plays a significant role in alleviating stress, regulating negative emotions, and promoting physical and mental health. Many tennis enthusiasts have reported that the distinctive sound and rhythm of hitting a tennis ball are important factors that contribute to their addiction to the sport. This particular sound can effectively alleviate their work-related stress, life pressures, and negative emotions, providing them temporary respite from feelings of frustration, anxiety, sadness, or anger. Even watching or listening to the game from outside the court can induce a relaxed and enjoyable feeling. This suggests that the impact of tennis on anxiety may not solely be attributed to the increase in physical activity levels; as the unique sound of the sport may also play a contributory role in relieving anxiety. This article validated this hypothesis and analyzed the acoustic characteristics of tennis strokes.

## 2 Materials and methods

### 2.1 Anxiety coefficient calculation

This study employed the “Zung Anxiety Self-Assessment Scale” (SAS, Zung, 1971). The anxiety coefficient (*q*) was calculated by adding up the scores of all the questions and dividing by the total possible score. The formula was calculated as:


$$q=\frac{\sum_{i=1}^{n}a_{i}}{T}$$

For each question *i*, the score is recorded as *a*, where *n* (the total number of questions) is 20 and *T* (the total marks on the paper) is 80.

The anxiety coefficient (*q*) is typically rounded to two decimal places in practical applications, but for this experiment, the exact value was retained to obtain a more detailed variation. According to the results of testing based on Chinese norms, if *q* ≤ 0.5, it represents a normal level of anxiety; *q*∈(0.5,0.59) represents mild anxiety; *q*∈(0.6,0.69) represents moderate anxiety, and *q*∈(0.7,1) represents severe anxiety.

### 2.2 Participants and power calculation

This study recruited volunteers from the Psychological Counseling Center of West China Normal University. In order to recruit suitable volunteers, 368 participants who self-reported being in a state of long-term anxiety filled out the SAS and several other questionnaires to disguise the research intent (Including preferences in sports, music, reading, entertainment methods, as well as sources of anxiety and stress). As mentioned in the previous section, moderate anxiety can help people stay alert and improve their work or study efficiency. So, the inclusion criteria required an *q* ≥ 0.6. Exclusion criteria included: (1) a history of any psychotropic medication use; (2) diagnosed emotional disorders by doctor; (3) significant hearing impairments (Pure tone audiometry > 25dBHL); (4) having a certain level of knowledge or experience in tennis (Tennis practitioner and enthusiast).

After calculation, this study is expected to require 90 (*n* = 90, *n*_1_ = *n*_2_ = 45; *f* = 0.3, *α* = 0.05, 1–*β* = 0.80; from G*power 3.1.9.7; Faul et al., 2007, 2009) experimental group participants to obtain appropriate results. After screening, we identified 96 (*n* = 96; Males = 37, Females = 59) eligible subjects and randomly divided them into two groups: experimental group and control group (*n*_1_ = *n*_2_ = 48).

### 2.3 Experimental design

**Figure 1:**
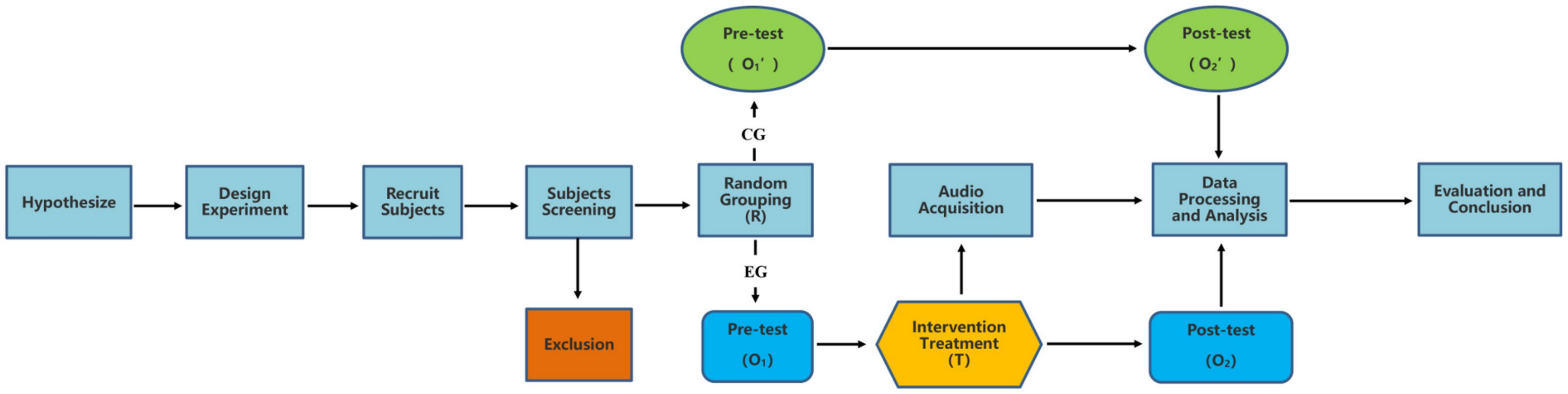
Experimental flow diagram.

Since the primary focus of the study was to examine whether the sound of tennis balls striking affects levels of anxiety, both qualitative and quantitative research methods were employed simultaneously. The experimental section employed a pre- and post-test completely randomized design; this is the quantitative research section. The design features are as Figure 1: R represents the subjects randomly assigned to two groups, O_1_ represents the pre-test score, O_2_ represents the post-test score, and T represents the Intervention experiment.

### 2.4 Procedure

#### 2.4.1 Intervention experiment

Due to constraints imposed by the size of the venue, participants in the experimental group were divided into two groups of 24 individuals each. They sequentially entered the venue to conduct the experiment. The subjects were located near the umpire’s chair with their backs to the court and their eyes closed to avoid visual stimulation. They meditated for 1 min to calm down before the sound stimulation began. Then, the experimenter started the tennis match without making any extra noise, and the subjects were asked to participate in the sound stimulation for 5 min. Approximately 150–200 hitting sounds were heard within a five-minute period. After the experiment concludes, participants are required to additionally complete some simple test question, describing subjectively whether they perceive a relief in anxiety and selecting the tennis ball striking sound that they find more pleasant (either a crisp hitting sound or a striking sound with friction). This is the qualitative research section. During the 4 weeks of the experiment, the subjects were required to maintain their daily routine and avoid additional exercise or recreational activities. The first SAS test was conducted within 3 days prior to the start of the experiment, and the second SAS test was completed within 3 days after the conclusion of the experiment.

#### 2.4.2 Sound capture

A clear and windless day was chosen to measure the sound of consistent batting, including hits with strong spin (such as serves and forehands with spin) and hits with very little spin (such as flat serves and flat forehands), at the umpire’s chair on an outdoor tennis court.

#### 2.4.3 Materials and processing equipment

Tennis Racket: Wilson WR120211U2-PRO STAFF97 V13.0 (Parameters: Empty weight 315 g, Face size – 97 square inches, Threaded pounds - 52 Pounds, Threading mode: 16 × 19). Tennis: Slazenger Wimbledon - 340940. Venue: Outdoor standard hard surface. Recording equipment: RODE Video Micro II. Recording software: Adobe Audition 2022. Audio analysis software: Adobe Audition 2022.

### 2.5 Ethics, transparency and openness

This study’s methodology is explicitly described in the methods section. All data and materials used in the study are available upon request, including samples, measurement methods used, executed statistical analyzes, raw data, and any other materials used in the study. This study strives for transparency in reporting its findings. All survey results are reported in a clear and concise manner, with appropriate descriptive statistics and effect sizes provided where relevant. The limitations of the study and recommendations for future research are discussed at the end of the paper. This study strictly adheres to the APA ethical guidelines; including obtaining informed consent from participants and protecting their privacy and confidentiality. All the eligible participants voluntarily participated. All participants read and signed an informed consent form. This study received support from the Ethics Committee (Attached review form).

## 3 Result

### 3.1 Intervention experiment data and analysis

**Figure 2:**
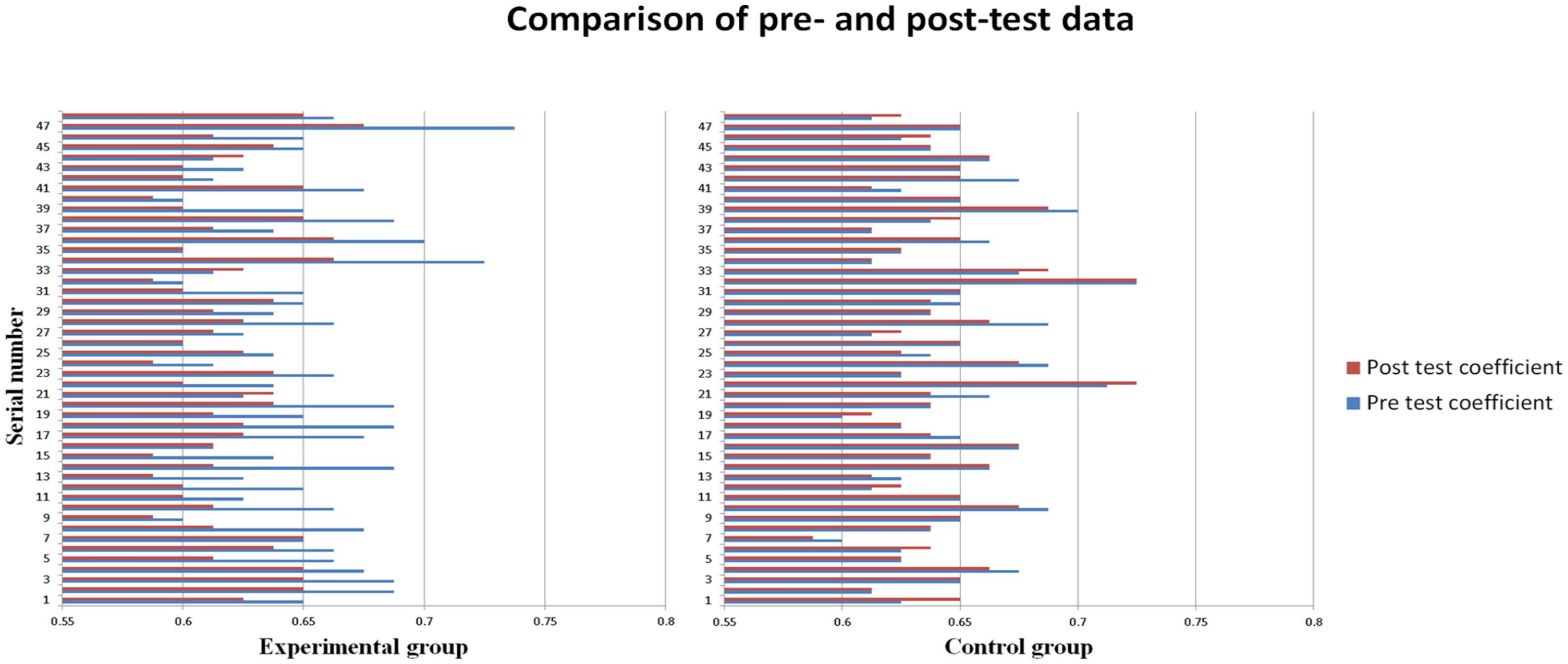
Comparison of experimental group and control group.

Figure 2 shows the basic situation of all subjects. As can be seen from Figure 2, the anxiety coefficients of the subjects in the experimental group showed different degrees of reduction after the intervention experiment. In contrast, the control group subjects basically maintained their original anxiety level, and the overall change was not significant. Statistical tests were carried out on the samples to obtain more detailed results of the data analysis (See Tables 1 2).

**Table 1:** Descriptive statistics of outcomes of intervention experiments.

Measure		M*	SD*	SE*	B(95% CI)	Min	Max
EG*	Age	20.79	1.688	0.244	(20.30, 21.28)	18	24
	Pre-test score(O_1_)	51.98	2.638	0.381	(51.21, 52.75)	48	59
	Post-test score (O_2_)	49.67	1.860	0.269	(49.13, 50.21)	47	54
	Pre-test coefficient(*q_1_*)	0.649740	0.0329702	0.0047588	(0.640166, 0.659313)	0.6000	0.7375
	Post-test coefficient(*q_2_*)	0.620833	0.0232547	0.0033565	(0.614081, 0.627586)	0.5875	0.6750
CG*	Age	20.60	1.594	0.230	(20.14, 21.07)	18	23
	Pre-test score(O_1_′)	51.69	2.299	0.332	(51.02, 52.35)	48	58
	Post-test score (O_2_′)	51.56	2.153	0.311	(50.94, 52.19)	47	58
	Pre-test coefficient(*q*_1_′)	0.646094	0.0287316	0.0041471	(0.637751, 0.654437)	0.6000	0.7250
	Post-test coefficient(*q*_2_′)	0.644531	0.0269093	0.0038840	(0.636718, 0.652345)	0.5875	0.7250

*EG, Experimental Group; CG, Control Group; M, Mean; SD, Standard Deviation; SE, Standard Error.

**Table 2:** Results of one-way analysis of variance.

Measure		SS	df	MS	F	p*	η^2^*
Pre-test coefficient	Between	0.000	1	0.000	0.334	0.565	0.000
	Within	0.090	94	0.001			
	Total	0.090	95				
Post-test coefficient	Between	0.013	1	0.013	21.311	0.000	0.178
	Within	0.059	94	0.001			
	Total	0.073	95				
Coefficient variation	Between	0.018	1	0.018	61.490	0.000	0.400
	Within	0.027	94	0.000			
	Total	0.045	95				

*When *p*(Sig.) < 0.001,shown as 0.000(*p* is the level of significance, when *p* < 0.05, the correlation holds, and vice versa the correlation does not hold).

*η^2^ is Effect size.

Based on the descriptive information(For Table 1), it can be observed that the age difference between the experimental group (EG) and the control group (CG) is small, and there is no significant age difference between the two groups [EG: M_Age_ = 20.79, SD = 1.688, 95% CI = (20.30, 21.28); CG: M_Age_ = 20.60, SD = 1.594, 95% CI = (20.14, 21.07)].

The pre-test scores and coefficients of the EG [O_1_: M = 51.98, SD = 2.638, 95% CI = (51.21, 52.75); *q*_1_: M = 0.649740, SD = 0.0329702, 95% CI = (0.640166, 0.659313)] and CG [O_1_': M = 51.69, SD = 2.299, 95% CI = (51.02, 52.35); *q*_1_': M = 0.646094, SD = 0.0287316, 95% CI = (0.637751, 0.654437)] are similar, indicating no significant differences in the pre-test measures between the two groups.

However, the post-test scores and coefficients of the EG [O_2_: M = 49.67, SD = 1.860, 95% CI = (49.13, 50.21); *q*_2_: M = 0.620833, SD = 0.0232547, 95% CI = (0.614081, 0.627586)] are significantly lower than those of the CG [O_2_': M = 51.56, SD = 2.153, 95% CI = (50.94, 52.19); *q*_2_': M = 0.644531, SD = 0.0269093, 95% CI = (0.636718, 0.652345)]. In contrast, there are more pronounced differences in the post-test results.

The analysis of variance (For Table 2) reveals the pre-test scores show minimal differences between different groups (SS = 0.000, MS = 0.000, df = 1, *F* = 0.334, *p* = 0.565, *η*^2^ = 0.000). The within-group differences in pre-test scores are also relatively small (SS = 0.090, df = 94, MS = 0.001). This indicates that the experimental and control groups had similar levels of anxiety before the intervention experiment.

The between-group differences in post-test coefficients are significant (SS = 0.013, df = 1, MS = 0.013, *F* = 21.311, *p* = 0.000, *η*^2^ = 0.178), while the within-group differences are relatively small (SS = 0.059, df = 94, MS = 0.001). This suggests that the differences in post-test coefficients primarily arise from different groupings, with the experimental group showing a significant decrease in post-test coefficients.

The between-group differences in coefficient variation are also highly significant (SS = 0.018, df = 1, MS = 0.018, *F* = 61.490, p = 0.000, *η*^2^ = 0.400), whereas the within-group differences are relatively smaller (SS = 0.027, df = 94, MS = 0.000). This further confirms the previous findings.

### 3.2 Batting sound data and analysis

**Figure 3:**
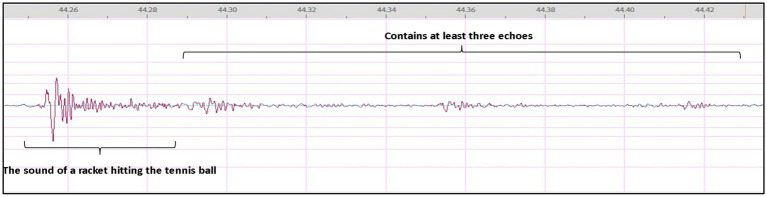
Waveform diagram of the sound of a complete stroke.

To enhance the authenticity of the experimental setting, an extensive corpus of audio data was collected at the outdoor venue, capturing the distinct waveforms of each individual strike. Subsequently, a noise reduction process was performed on the audio data to yield a more precise waveform graph. It is shown in the following picture (Figure 3).

**Figure 4:**
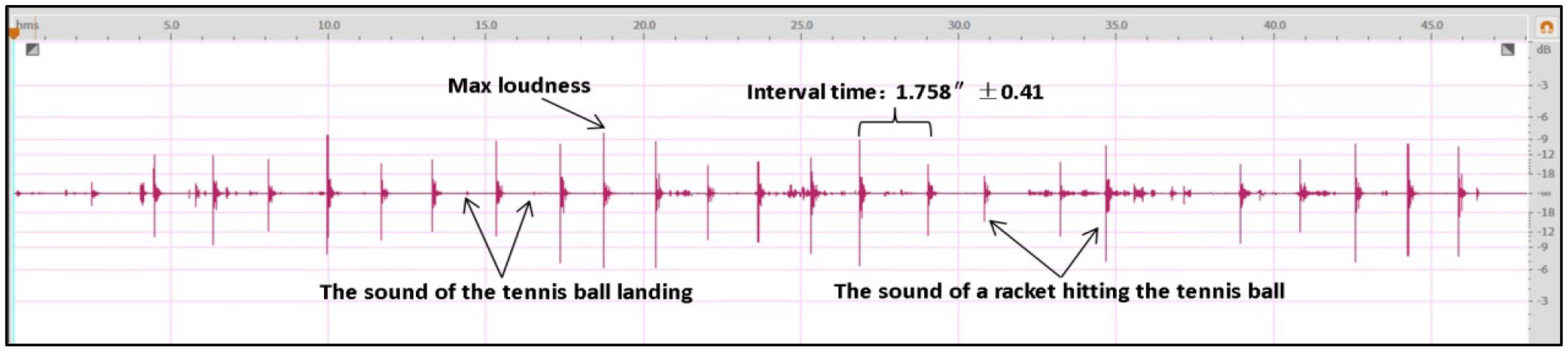
Waveform graph of a multi-round strike.

The regular waveform depicted in the graph displays the fundamental characteristics of the sound produced by a tennis stroke, exhibiting a smooth and uniform pattern. A complete stroke lasts for no more than 0.2″ and encompasses at least three distinct sound levels, implying that the sound of a tennis ball is comprised of a combination of multiple sounds in rapid succession, giving rise to a rich and resonant stroke sound. To provide a more consistent and accurate representation of the multi-round batting sound data, the original audio was processed further. The results are shown in the figure below (Figure 4).

Based on the sound data, it can be observed that the sound intensity of the racket hitting the ball, as perceived from the umpire’s stand, falls within the range of (−3.5 dB to −9.5 dB), while the sound produced when the tennis ball hits the ground is approximately -21 dB. The average interval between successive racket impacts is 1.758″ (1.758″ ± 0.41).

**Figure 5:**
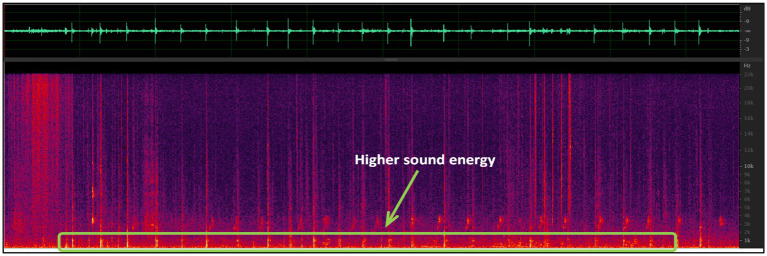
Original audio spectrum analysis graph.

Moreover, spectral analysis of this relatively regular audio data yielded the following results (Figure 5).

**Figure 6:**
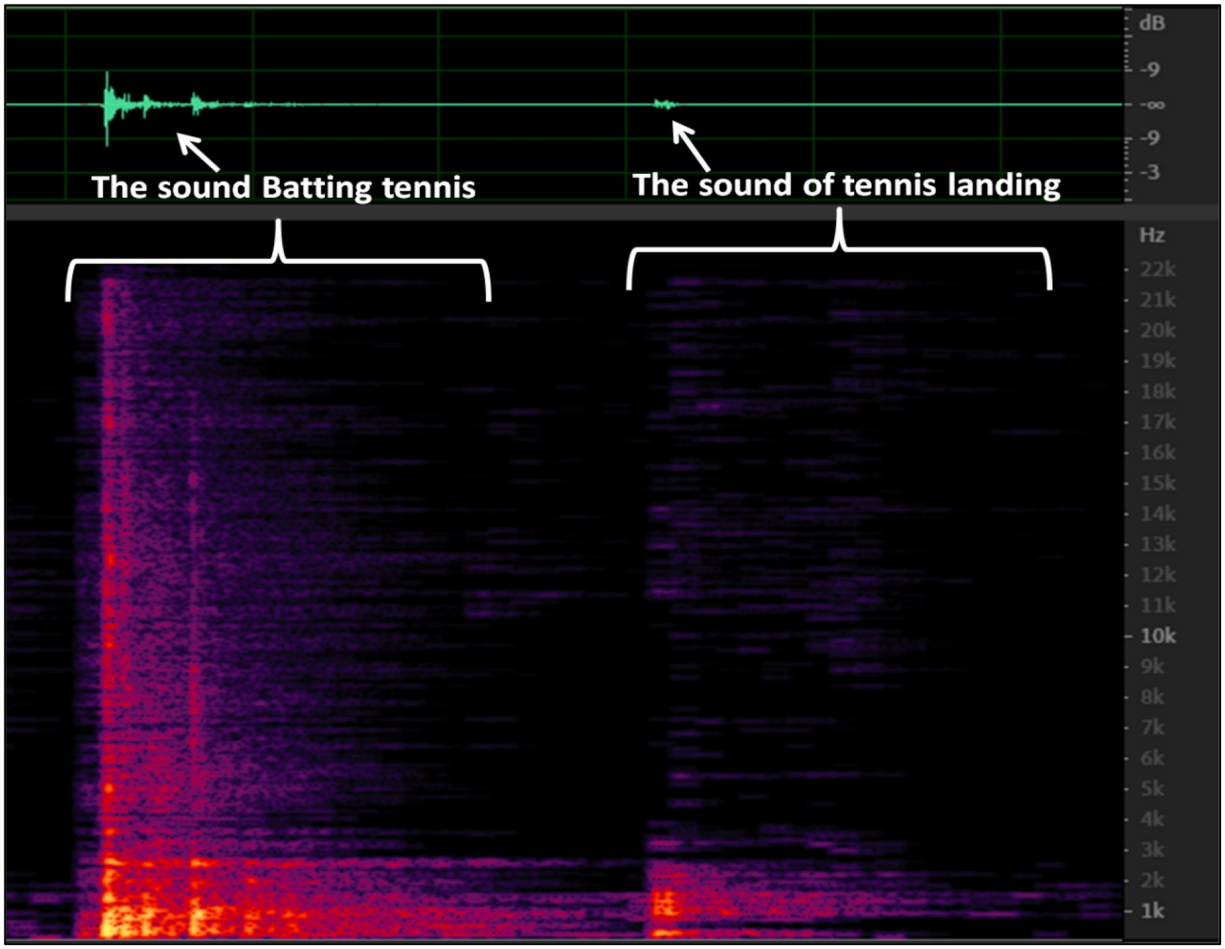
Comparison of the sound of the racket hitting the ball and the sound of the tennis ball hitting the ground.

The spectrograms demonstrate that the sound produced by hitting a tennis ball corresponds to a broad spectrum of frequencies that extend beyond the range of 0–22 kHz. However, the primary energy is concentrated in the range of 0–3,000 Hz, which is reflected in the bright colors in this frequency range, suggesting that the lower frequencies contribute the most energy. Upon comparing the waveform and spectrogram, it was observed the sounds with the highest amplitudes occur within the 0–3,000 Hz range. To obtain clearer results, noise reduction techniques were applied to the audio due to the substantial amount of ambient noise present in the recording. Presented below is a sample of the most representative data obtained (Figure 6).

**Figure 7:**
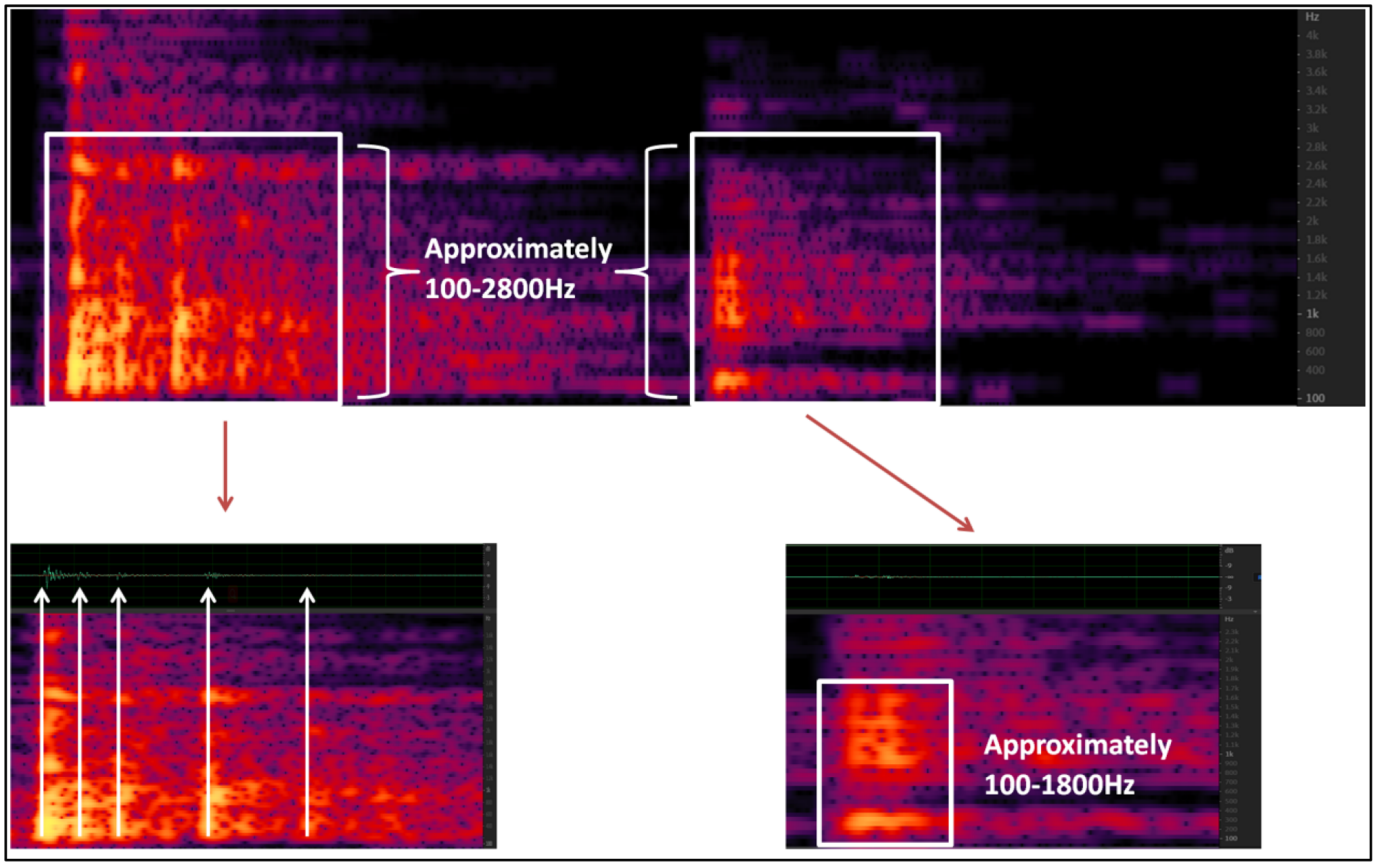
Main frequency distribution ranges for tennis ball strikes and tennis ball landings.

The diagram displays the audio waveform of the racket hitting the ball on the left and the sound of the tennis ball making contact with the ground on the right. The left side of the diagram exhibits a wide range of frequencies, with the peak energy being concentrated in the lower frequencies. Conversely, the sound of the tennis ball hitting the ground lacks high frequency data, implying that the tennis ball produces a low frequency sound. Figure 7 provides a magnified image that reveals more intricate details.

As depicted in Figure 7, the energy density of the sound is highest in the frequency range of approximately 100–1800 Hz, which corresponds to the fundamental sound of the tennis ball. Additionally, the overtone component of the tennis ball is present within the range of 1800–2,800 Hz, audible both when the racket hits the ball and when the ball hits the ground.

## 4 Discussion

The significant differences observed in the post-test coefficients and coefficient variation between the experimental and control groups indicate that the sound intervention has an impact on these two measures, providing significant explanatory power in reducing anxiety levels.

In summary, the results of the analysis suggest that the pre-test scores did not differ significantly between the experimental and control groups, indicating similar levels of anxiety before the intervention. However, the post-test coefficients and coefficient variation showed significant differences between the groups, indicating the effectiveness of sound intervention in reducing anxiety levels.

The experiment yielded notable findings on the effect of sound stimulation from tennis ball hitting on the anxiety level of subjects in the experimental group. Specifically, the results showed a reduction in anxiety levels with a mean anxiety coefficient decrease of 0.02896, even in the absence of physical activity. Nevertheless, the anxiety reduction was not significant enough to fully alleviate the anxiety of the subjects, with some of them transitioning from moderate to mild anxiety. In contrast, the control group exhibited a minimal mean anxiety coefficient change of only 0.00156, indicating little improvement in their anxiety status. The comparison of the two groups revealed that the anxiety coefficients of the experimental group decreased significantly, implying that the sound of tennis ball hitting is a contributing factor in reducing anxiety, not just the physical activity itself. The findings suggest that tennis, with its unique sound, possesses a soothing effect on anxiety.

After the intervention, the majority of the subjects who were interviewed reported that the sound of the tennis ball was perceived as crisp., full, and evenly spaced, akin to a rhythmic drumbeat. Among the different strokes, the sound of flat serves and flat strokes was found to be the most pleasant, while the cutting or strongly spinning balls were perceived to have varying degrees of friction, which, although still comfortable overall, was less than ideal compared to the crisp hitting sound that is a hallmark of tennis and one of the reasons why many enthusiasts are enamored with the sport. To gain insight into the fundamental properties of this sound, the recorded sound of a tennis ball being struck was analyzed.

The wide frequency range contributes to the dominant perception of the tennis sound as a deep bass with a subtle high-end crispness. This sound is composed of a mixture of decaying sounds within a short period of 0.2″, resulting in a rich and complex overall sound. The sound created when hitting the ball is a composition of vibrations originating from the racket, string, and the tennis ball itself. The sound above 2,800 Hz is likely a combination of vibrations from the tennis string and tennis racket. This finding further supports the previous assertion that a complete tennis stroke sound is a combination of multiple echoes occurring within a short period, which contributes to its perceived fullness. However, the new spectral analysis indicates that there are more than three distinct levels of sound that contribute to this full stroke. It should be noted that these results were obtained using the available equipment, and with more advanced equipment, more precise data could potentially be obtained.

The temporal structure of the sound of multiple rounds of tennis is governed by the rules and regulations set forth for the game and specifications of the court. This characteristic temporal structure engenders a rhythmic auditory experience for the observer. However, the temporal structure varies depending on the level of the player and the velocity of the ball. As the ball velocity increases, the time intervals between the shots decrease, leading to a tighter and more precise rhythm. Specifically, for an average ball speed of 93.6 km/h (26 m/s), assuming the player stands approximately 1 m away from the baseline when hitting the ball, the interval between shots is approximately 1 s. The rhythmic pattern generated by multiple rounds of tennis is reminiscent of the beat of popular music, albeit achieving such a tempo necessitates exceptional levels of athletic prowess and control. Professional tennis players are perhaps more inclined to attain and maintain such a rhythm during matches. The rhythmic nature of the sound produced by the game adds to its allure for spectators of professional tennis events.

Research has demonstrated that rhythmic sounds can be comforting (Zimba and Robin, 1998; Iversen et al., 2008) as the human brain has a basic predictive function for rhythmic sounds (Bubic et al., 2010). In the same way that people listen to music to anticipate the next point of repetition, sounds with varying intensities and rhythms can impact human auditory organization and evoke diverse emotions (Koelsch, 2014; Simon et al., 2017). A stable sound rhythm allows the brain to predict the next sound node, thereby creating a sense of order and coherence.

While a soothing musical rhythm can relax the mind and body and relieve stress, fast-paced music can be exciting; however, sounds that are too fast can also induce panic and stress, such as sirens, screams, and mechanical noise from construction sites (Nakajima et al., 2016). Another study suggests that rhythmic visual and auditory stimuli lead to high levels of activity on the right side of the human brain (Brauchli et al., 1995), which may explain why people enjoy the sound of tennis.

The frequency range of 100–5,000 Hz is widely recognized as the most sensitive range for human perception of sound (Masterton et al., 1969; Brant and Fozard, 1990; Marquardt et al., 2007; Salminen, 2015). However, sounds above 4,000 Hz can lead to hearing loss (Dreisbach and Siegel, 2001; Sarrou et al., 2018), and sounds of appropriate frequency and amplitude are generally more responsive and comfortable (Hung and Dallos, 1972; Salminen, 2015). Tennis sound frequencies fall within the range of the most sensitive sounds for humans, which explains why most people find the sound of tennis pleasing to listen to. This pleasant and rhythmic sound is an important reason why it is enjoyed by many.

However, it is not only soothing music that can influence human emotions. For example, the sound of a hi-hat cymbals (a metallic percussion instrument commonly used in drum kits) may not be pleasant on its own, but when combined with other instruments it contributes to many popular music genres. Similarly, the sound of a basketball hitting the ground may not be very pleasant on its own to people who do not normally exercise, but during an intense game, the sound can be quite exciting, possibly due to the combined visual and auditory stimuli. Various sports have their own distinctive sounds, such as basketball, football, volleyball, badminton, and table tennis, which contribute to the unique attributes of the sport itself. Numerous studies have explored the combination of sound and sport, applying different music to improve athletic performance or enhance recovery before, during, and after competition (Curran, 2012; Karageorghis et al., 2012, 2013; Karageorghis and Jones, 2014; Smirmaul, 2016; Stork and Martin Ginis, 2017; Terry et al., 2020). These studies suggest that appropriate sound stimulation combined with exercise can lead to better training, competition, and recovery outcomes.

The various movement stimuli may not affect various brain structures to the same extent. For example, sound stimuli of movement may stimulate more temporal lobe and amygdala regions (Frühholz et al., 2015; Zhao et al., 2018; Manno et al., 2019), while visual stimuli would stimulate more visual cortex and occipital lobe (Masters et al., 2015), and limbic activity may stimulate the cerebellum and striatum more (Sosa et al., 2015; Miterko et al., 2019), among others. Existing studies have typically analyzed exercise in general and have not examined the weight of sound stimuli in the various mechanisms by which exercise affects mood. Most studies have tended to focus on the physiological changes brought about by changes in exercise intensity but have neglected the effects of the sound of exercise on the body. The present study has improved the understanding of the effect of sound on human emotions during exercise, which is perhaps what distinguishes various sports from running alone. It also shows that the mechanisms by which exercise affects the human body are more complex.

## 5 Conclusion

The experiment confirmed the previous conjecture, the sound of tennis alone has a significant anxiety-reducing effect on subjects, with an average anxiety factor reduction of 0.0289, and some subjects even reported a decrease from moderate to mild anxiety. The sound produced by the impact of a tennis ball encompasses a wide frequency range, resulting from the combination of sounds generated by the racket, strings, and the ball itself. This amalgamation creates a complete and pleasant sound, with the low frequencies providing depth and the high frequencies contributing to crispness. The sound exhibits a continuous and uniform rhythm, accompanied by variations in pitch, enhancing its enjoyable qualities. This auditory profile is well-suited to the human auditory system. All this indicates that the alleviation of anxiety in tennis is not solely derived from the elevation of physical activity levels. The distinct and soothing sound it produces also serves as one of the contributing factors to anxiety relief.

## 6 Limitations and future directions

This paper investigates the effect of tennis sound on anxiety, confirming the anxiety-reducing and mood-regulating effects of tennis stroke sounds, and examining the fundamental properties of this sound. The application of Fourier transform to convert acoustic signals into visual images greatly aids this study. Although the algorithms used by the latest audio analysis software are highly advanced and deviations from actual data are already minimal, professional audio analysis instruments can still provide more accurate results. Better equipment may also be able to uncover more details. The sound generated by exercise is a significant aspect of movement, and the impact of these sounds on the human brain requires verification through more specialized medical or biological experiments. Improving this aspect of research is expected to further enhance the understanding of the mechanisms through which exercise affects the human body.

## Data availability statement

The original contributions presented in the study are included in the article/Supplementary material, further inquiries can be directed to the corresponding author.

## Ethics statement

The studies involving humans were approved by Ethics Review Committee of China West Normal University China West Normal University. The studies were conducted in accordance with the local legislation and institutional requirements. The participants provided their written informed consent to participate in this study.

## Author contributions

HW: proposed the theory, viewpoint, method, data processing, and wrote the paper. GZ and XL: made corresponding contributions to the experiments and data collection in the paper. SHP guided the paper. All authors contributed to the article and approved the submitted version.
